# “A place without walls, only opportunities”: personal accounts of attending Recovery Colleges in Norway

**DOI:** 10.3389/fpsyt.2023.1233598

**Published:** 2023-10-25

**Authors:** Anne Schanche Selbekk, Linda Teie Kvelland, Rebecca Nordås, Aasa Kvia, Inger Eide Robertson

**Affiliations:** ^1^Department of Public Health, The Faculty of Health Sciences, University of Stavanger, Stavanger, Norway; ^2^Mestringsenheten, Sandnes Municipality, Sandnes, Norway; ^3^The Regional Competence Centre on Alcohol and Drugs Prevention - Stavanger (KORUS Stavanger), Stavanger, Norway

**Keywords:** recovery college, qualitative methods, citizenship, stigma, positioning theory

## Abstract

**Introduction:**

Recovery colleges (RCs) are learning environments, first established in the UK, based on principles that support positive life changes and reduce stigma related to challenges with mental health and substance use problems. RCs offer courses based on co-production processes and are designed and delivered jointly by individuals with lived experience and professional experts. The courses are open to anyone, attracting people with a variety of life experiences. RCs are non-clinical environments that provide individuals with the identities of students and/or trainers as autonomous and independent agents. In this paper, we investigate experiences of being a part of a RC in Norway, either as a student and/or as a course trainer with lived experiences of mental health or substance use challenges. We ask the following research question: What kinds of personal and social processes are enabled by being part of a recovery college from the perspective of persons with experience-based competence?

**Materials and methods:**

The study is qualitative and explorative based on 11 individual (*N* = 11) and two focus group interviews (*N* = 8). Participants were recruited from two of the first RCs in Norway between August 2021 and January 2022.

**Results:**

Study participants describe how their involvement in a RC provided them with opportunities to assume new positions in their recovery process, both related to former institutional identities given in the course of treatment and related to the way they see themselves as people struggling with mental health and substances use challenges. Attending a RC represented significant transitions (1) from an institutional position as “sick” or as “what’s on the paper” into a position as “a whole person”; (2) from being in in a position as a recipient of care to the position as actively responsible for life changes; (3) from seeing themselves as worthless to seeing themselves as persons with resources; (4) from being alone to being part of a fellowship. Participants describe being part of a RC as an invaluable addition to other kind of support or help.

**Discussion:**

It is important to provide alternative arenas like RC for facilitating work with life changes, as an invaluable addition to regular services.

## 1. Introduction

Recovery from mental health issues or substance use problems is complex processes that extend the scope of traditional health services and is closely embedded with personal meaning making and social reintegration (1). Barriers in the processes revolve around societal stigma (the way other categorize you), self-stigma (the way you internalizes a societal stigma) and shame associated with these issues or conditions (2, 3). Recovery can be understood from various perspective, including a “clinical” approach that emphasizes symptom reduction and relapse prevention, and a “personal” and “social” approach that focus on personal goals, individual strength and social reintegration (4). The latter perspective on recovery have increasingly influenced the delivery and organization of services in the field of substance use and mental health, particularly in community services, but also within specialist health care services (5, 6). An optimal continuum should encompass both biomedical treatments and community-based, person-centered approaches that emphasize personal and social recovery (5).

Recovery Colleges (RCs), as part of a wider movement of personal and social recovery philosophy and recovery-oriented practice, are learning environments based on principles that aim to support positive life changes and reduce stigma related to challenges with mental health and substance use issues (7). RCs offer courses based on co-production processes that include persons with lived experiences and professionals, and are open to everybody across life experiences, providing identities as students/trainers to persons as autonomous and independent agents, and in a non-clinical environment (8, 9). A noted strength of RCs is that they create an alternative space where a culture of co-production can emerge more readily than in traditional mental health services or conventional peer-led support groups and organizations (10, p. 41). Internationally, 221 RCs are now identified across 28 countries in five continents (11). RCs are now established in at least 20 countries across the world (12). In UK only, 88 colleges have been initiated with some variation in organization, location (urban, rural), and other factors (13). Based on data from existing colleges, Hayes et al. (13) categorized these colleges in three clusters of characteristics, as either *strength-based* colleges, which are usually affiliated with specialist health care and based in health or social care buildings, or *community-based* colleges, which are almost exclusively unaffiliated with specialist care and based in community location, or as *forensic RCs* offered to forensic populations (13). As part of an enhanced emphasis on personal recovery and recovery-oriented approaches in Norway, the first three colleges, were introduced in 2019 inspired by the RC in Nottingham, and since then additional RCs are established or on their way to be established in different geographical areas. RCs in Norway are collaborating nationally and internationally to ensure the quality criteria and fidelity of the courses they design and deliver (14, 15).

How does involvement in a RC affect an individual’s personal recovery process? Several studies, including one from Norway, have focused on various aspects of RCs, and a growing body of evidence indicates high levels of satisfaction among students with challenges related to substance use and/or mental health issues regarding attainment of recovery goals, improved quality of life and well-being, increased knowledge and self-management skills, and reduction of service use (16–19). A qualitative review focusing on student outcome and experiences demonstrated that the colleges represented a shift in power away from the traditional roles of clinicians/patient to other relational dynamics, and that participants adopted the role of students (20). The social benefits of attending RCs were emphasized by many (but not all) participants, and the colleges were seen as facilitating personal growth (20). Questions about when an RC may be of most benefit to the recovery process, and what happens after a course is over, have also been discussed (20). To our knowledge there has only been two previous peer-review study of RC initiatives in Norway (16, 21). A RC is founded on the principles of co-creation between professionals and individuals with lived experience, both in the design and delivery of courses. However, in this study, we will exclusively emphasize the perspective of individuals with lived experience either as students or as trainers to explore aspects connected to their personal recovery journeys. Drawing from a Norwegian sample of participants at RCs with life experiences of mental health or substance use challenges, we ask the following research question: What kinds of personal and social processes are enabled by being part of an RC from the perspective of persons with experience-based competence?

## 2. Materials and methods

The study is qualitative and explorative based on semi-structured interviews (*N* = 11) and two focus group interviews (*N* = 8) with two categories of study participants (see Table 1). The participants in the individual interviews were *students* attending RC courses, recruited from one RC in Norway. The participants of the focus group interviews were *course trainers*, involved in co-producing and co-leading RC courses, and were recruited from two different RCs in Norway. The interviews were conducted as part of an evaluation study of RCs in western Norway from August 2021 to January 2022. Common to all the participants were experience of some kind of substance use or mental health issue in their lives and encountered health or social services in the context of those issues. Still, the study contained no specific questions regarding diagnosis. We opted to include two different participants groups to broaden the scope of our research question, seeking perspective from both students and trainers with lived experience. While they occupy different roles within the RC and are at various stages in their recovery process, all share personal experience related to substance use and mental health challenges. Furthermore, with the exception of one trainer, all trainers also have previous experience as students themselves. As a result, we contend that combining the experiences of these two groups in a collective analysis would be beneficial.

**Table 1:** Overview over study participants (*N* = 21).

	Students of experience (from one RC)	Course trainers of experience (from two RCs)
Data collection	Eight individual interviews (*N* = 8)	Two focus groups with four participants in each group, + three individual interviews (*N* = 11)
Gender	Men = 4 Women = 4	Men = 4 Women = 7
Age	20–29 = 1 30–39 = 3 40–49 = 1 50–59 = 2 60–69 = 1	Information not available
RC course attended/led	1 course = 4 2–3 courses = 4	All course trainers have been former course participants All course trainers have experiences of facilitating/leading two courses or more
Experience		All course trainers, except one, have both experiences with developing and facilitating/leading courses

In this study, we employed individual interviews and focus groups to collect the experiences of students and trainers, respectively. Individual interviews were chosen for students to delve into their personal processes concerning the course. For trainers, we conducted focus group interviews to facilitate a broader dialogue regarding their experiences in course leadership and their observations of group dynamics. During the focus group interviews, trainers seamlessly shifted between perspectives, discussing their role as course trainers and reflecting on their personal journey within the college, including their experiences as former students and their current role as trainers. Additionally, due to practical constraints, we conducted individual interviews with three trainers who could not participate in the focus groups.

Due to practical circumstances and capacity, the recruitment of *students* for individual interviews were limited to one college. A manager of the college sent out an e-mail to students (*N* = 22) who had participated in two or more courses to ask if they wanted to join the study. After several rounds of follow-up emails, four persons were recruited and contact information was passed on to Author 2. A new mail was sent out to a randomly picked selection of students who had participated in one course (*N* = 28). By this route four more persons were recruited for a total of eight participants, four woman and four men, 20–60 years old.

The recruitment of participants for the focus group interviews comprised the category *trainers*. These participants were recruited from two colleges, using the following procedure: Author 3 sent an e-mail with all the necessary information to managers of the two colleges, who forwarded it to the college’s trainers. Those who wanted to participate then contacted Author 3 and focus group, and individual interviews were conducted. In total two focus-group interviews and three individual interviews with 11 participants, seven woman and four men, were conducted.

We prepared two separate interview guides for the two participant categories. However, the data used for this study’s analyses were derived from the following common themes we selected: how individuals established contact with RC, their expectations of participating in RC, their experiences with course participation and co-creation of courses, group dynamics and social connections within courses, and the perceived value and utility of RCs (22, 23). Additionally, students were questioned about their recovery process and experiences with traditional services, while trainers were asked about collaboration in courses between trainers with lived experience and those with professional backgrounds. The interview guide was developed without any specific theoretical foundation.

When it comes to fidelity, both colleges align with the seven non-modifiable components that define a RC and distinguish them from other forms of treatment and support: valuing equality, promoting a culture of learning, tailoring support to individual students, co-producing content with the RC community, fostering social connectedness, maintaining a community focus, and committing to the recovery process (10, 14, 15). Recent reports show how Norwegian RCs in general have a high fidelity score (11). All courses are co-created by healthcare, social or other professionals in collaboration with individuals who have lived experience. Additionally, apart from one course, all courses are jointly led by experts of experience and professionals. The one course program exclusively peer-led, falls under the organizational umbrella of one of the study’s RCs, representing two out of the 55 courses offered between 2019 and 2022. Regarding the modifiable components — availability to all, locations, distinctiveness of course content, strength-based approach, progressive nature (10)— the two colleges involved in this study can be characterized as community-oriented RCs. They are situated in community settings with a strong emphasis on fostering social connectedness (13). One of the colleges has close cooperation with local health institutions and works in a de-centralized rural environment, but with a common base in a more centralized location, a type of college that is like what Muir-Cochrane et al. (24) call a “hub and spoke” model. The other college is centered around a physical building. Both RCs are established as part of a strategy focusing on recovery-oriented community mental health and substance use services. Participation in the RC is, in principle, open to all. Furthermore, there are no restrictions on course content. It is also specified in the quality criteria that courses do not serve as a substitute for regular treatment or formal education (15).

Courses offered in these settings are typical focused on personal and social recovery processes, coping, managing everyday life and art. The courses normally last from 4 to 7 weeks, with weekly (or twice a week) meetings lasting from 1.5 to 3 h each, and they have small classes consisting of 8–15 people. All courses involved in-person meetings that are organized as a combination of short lectures, reflections and sharing of experiences in plenary sessions, individual tasks, and group work. Upon completing a course, participants receive a certificate documenting their participation. However, these courses are not part of any formal education program. Many of the students follow several different courses. One of the colleges has extended activities like “open house,” for people to gather at certain times, and open lectures. All citizens are welcome to join the college – all that is required is that one is willing to learn new things about coping in everyday life. Persons with substance use backgrounds and mental health issues mix in the courses. From 2019 to 2022, the two colleges jointly provided a sum of 100 separate courses, spanning 16 unique course concepts, with a total enrolment of 1,020 students (25). The RCs are funded as projects by grants from local, regional and national governments.

All interviews were tape-recorded and transcribed verbatim by Authors 2 and 3. The interview material is analyses according to the six stages of the stepwise-deductive induction (SDI) model from raw data to concepts/theories (26). The general idea is to work inductively upwards from data to concepts, and simultaneously conduct deductive tests downwards to check the contents and results on each stage. The six stages are generating data, processing data, empirical close coding, code grouping, developing concepts, and developing theory (26, p. 3). Transcription and empirical close coding and grouping of codes were carried out by Author 2 (8 individual interviews with students, 563 empirical close codes, 9 code groups) and Author 3 (3 individual interviews and 2 focus group interviews with trainers with lived experience, 600 empirical close codes and 14 code groups). Analysis were first done separately for each group by Author 2 (students) (22) and Author 3 (course trainers) (23).

The code group data from the two groups of study participants were then analyzed across groups by Author 1 in cooperation with the other authors, guided by the research question, creating new code groups and concepts, returning to the empirical close coding and data transcripts for verifying the cross-group analysis. The cross-group analysis of the data in this study primarily centered on the individual and social processes that RC courses enabled and facilitated for the participants. Future paper will delve deeper into the aspects related to the co-creation processes from the perspective of professionals and individuals with lived experience.

All the authors have backgrounds in social sciences, with an ontological focus and bias toward social mechanisms and processes. Author 1 and 5 hold academic backgrounds from sociology, while Author 2, 3, and 4 come from social work. However, the authors hold different institutional positions: Author 1 works at a university, Author 2 and 3 are former master’s students in Substance Use and Mental Health, now employed in community services, Author 4 is a Ph.D. student with ties to the municipality, and Author 5 works at a regional competence center on alcohol and drugs prevention. This diversity in academic backgrounds and practical experiences has provided a wide-ranging perspective for analyzing the material. The two former master’s students who collected the data began with limited prior knowledge about the RC, approaching their work with few preconceptions. In contrast, Author 1, 4, and 5 had more initial knowledge and familiarity with RCs.

To ensure privacy, the project is processed and recommended by the Norwegian Centre for Research Data (NSD, now SIKT) in accordance with applicable legislations (nr. 689,039 and nr. 660,505). The Data Protection Officer at the University of Stavanger appraised that the project was properly carried out, and that benefits and risks are assessed against each other in accordance with the Guidelines for Research Ethics in the Social Sciences and the Humanities (27). The project was not considered for ethical approval at the Regional Committees for Medical and Health Research (REKs), as the project falls outside the scope of the Health Research Act from 2009.

The results from this study capture the accounts from persons with lived experience of participating in courses offered by a RC, and accounts from persons with lived experience of co-creating and co-facilitating/leading courses at the same arena.

### 2.1. Analytical framework

To capture the personal and social dynamics in being part of a RC, both the initial analyses and this new cross-group analysis (stage 6) were inspired by concepts from positioning theory (28). The principle behind positioning theory is that people, who both live within stories and are tellers of stories, are embedded in moral orders – that is, in public and private beliefs about the distribution of rights and duties to speak and act in certain ways (storylines), and the meaning and value of what is said and done (29, p. 268). Positioning theory holds that these beliefs about how rights and duties are ascribed make you either *position* yourself or *be positioned* by others in a certain way, within the repertoires of acts available, and in this way certain storylines are acted out in everyday encounters (29, p. 271).

Persons struggling with substance use and mental health issues may have encounters with health and social services or other kinds of support initiatives. If these encounters follow a traditional medical storyline, a person will be in a position as a patient with a condition who needs help from an expert. Institutional positioning occurs when an institution actively classifies people in ways that bring expectations of how they should function within that institution (28). Following this, individual perceptions and experiences of mental health and wellbeing must be viewed in the light of discursive content and strategies, referring to both the interpersonal and intrapersonal nature of positioning (30).

Positioning theory can also be defined as the discursive process in which rights and duties are taken up and laid down, ascribed and appropriated, refused, contested and defended in the fine grain of the daily encounters (29, 31, p. 132). As ongoing discursive processes, positions can be defined and renegotiated in any given interaction, and within one conversation or discursive context, several forms of positioning can take place simultaneously depending on what positions are available in that context (28). Persons can perform acts of repositioning by ascribing themselves alternative storylines that claim a different position [(32), p. 3; (33)]. This type of repositioning can also be seen as part of a healing process, in other words, as a way of repositioning “who we are” (31, p. 130).

In this study, we will use positioning theory as a starting point to illustrate and illuminate the experiences of persons with a background of substance use or mental health issues attending a RC.

## 3. Results

Most participants in the study reported that being part of RC were a positive experience, and for several participants, attendance contributed to life changing processes. The participants’ accounts can be connected to different types of inter- and intrapersonal processes of repositioning (28). The participants’ expectations before attending the RC were sometimes sceptical – can a course make me healthier? – and sometimes nervous, curious, unsure, challenging and demanding.

### 3.1. From being in a position as “sick” to being in a position of “a whole person”

Several participants contrasted their encounters with RC to former encounters with public health and social services. In the latter encounters, they described themselves as being positioned as “the sick person” (in terms of diagnosis), or as “what’s on the paper” (in term of convictions), and that they felt those categories or storylines, inherent in the encounters, were limiting and less useful to their current situation.

“…while at the treatment center you are met according to what’s on the paper, that is your verdict, this is what you have served your sentence for.” (Henrik, student)

“… that some well-educated people were telling me how I should do things, I am so terribly bored of that, and terribly bored of a system that only told me what I could not do, it made me even sicker [*sykeliggjorde meg så veldig*].” (Bendik, course trainer)

In the quotes above, the participants describe (previous) encounters with health and social services in which they were ascribed positions as “sick,” as “criminal,” entering into classic storylines of medical encounters between patient and doctor and legal encounters between offender and law enforcer. However, arriving at an RC they described themselves as entering into different positions, based in alternative storylines, as a whole person, as something other than a sick person:

“Here you are met as a whole person” (Henrik, student)

“You are not a sick person when you are here, You actually have something to give, and that it is heard, plain and simple.” (Heidi, course trainer)

Participants said that, in this environment, they could contribute and participate in the way they wanted without letting their background and history of illness define them. The focus was on learning and getting to know yourself better. This focus was considered very useful by the participants.

One of the participants noted that the physical place of the RC was less attached to a public stigma:

“So it’s not like if I was to walk into the alcohol and other drug clinic for example, or some other place that has a bit of stigma. I think it’s more embarrassing to go to the psychologist than I think it is to go into RC.” (Anne, student)

Nevertheless, the participants highlight how services have also evolved in recent years to adopt a more recovery-oriented and person-centered approach.

### 3.2. From being a passive recipient of care to being an active agent responsible for life changes

The participants also talked about the transition or repositioning from being passive recipients of help to being active agents who were responsible for life changes – from being in a passive position of a victim to being an actor in their own life. One participant noted that encounters with social and health services can traditionally get a person into a position where they get used to only receiving, not being an active agent yourself:

“… used to just receive, you just receive support, and then you can get into the role of a victim, a role where you just expect to receive. What you can do by being a course trainer here is that you transit from being in a victim role to giving something. And then very significant processes happen within people. (Bendik, course trainer)

Another participant corroborated this viewpoint, and noted specifically that there was nothing in the courses that put the participants in the position of victim.

“There was nothing that put us in a position of a victim. I’m against anything that puts one in a victim position, because it deprives one of responsibility and that’s not healthy. But it was just up and go, and I liked that.” (Bernt, student)

One of the main advantages of attending an RC is that all participants have chosen to be there, no one is “referred.”

“I do not like this referral thing. It takes the responsibility away from you. [Participating here] is on your own initiative. I think that is very good and very important. It is good to see that people in fact do it and participate. Because that is the way you can see changes and that is how you get better. […] This is available for everybody who wants it.” (Bernt, student)

It is interesting to observe how Bernt characterizes the act of referral as a means to shift responsibility away from the individual, in contrast to his experience at the RC. To be held responsible was highlighted as a significant factor for the experienced impact of the courses at the RC. Participants said that the focus on responsibility was a prerequisite for participating.

“Everything here is about […] everything is about ‘this is something I want to do’, and you are creating goals for yourself. It’s all up to you, there’s no one going to go in and fix you, in a way. You kind of have to… take charge and address it yourself.” (Emilie, student)

What one gains from participating in the courses depends on oneself, if one takes the responsibility and participate actively.

Participants said that they learned a lot about coping during the courses. The tools given in the courses were very hands-on, easily accessible and easy to use in everyday life. The course content was relevant and inspiring and gave them challenges and concrete tools so that they could implement what they had learned in their daily lives in a way that gave them a sense of coping. This, in turn, contributed to active use of these coping tools and made them want to learn more.

In this sense, the RC is an arena in which the participants can reposition themselves from being in need of care to being a normal citizen. Several of the participants said that prior to attending RC, they had been a place in life where they felt “lost” and without direction, and that the RC had helped them move forward in their recovery processes. They said that they had gained new direction and meaning in life.

### 3.3. From seeing oneself as a worthless to seeing oneself as a person with resources

Some of the participants mentioned that they had felt uncertain about whether the other students wanted to engage with them at all when they learned about their backgrounds.

“Because just before the course, I had a little thought of shame about what I had done and what my life was like. ‘Would others be interested in hearing about it?’ or ‘would others be interested in talking to me after hearing what I had done?’ So, I guess there were more bad expectations than good expectations, but those where more my own [bad] expectation to myself.” (Henrik, student)

Here a participant positions himself as inferior or an outsider. Participants talk about the process of getting “a new faith in oneself as a useful [resourceful] person” (expert of experience) and the process of “belief that I have worth” (student). The participants talked about how RCs facilitate these processes among the participants. The college is a place where you are a person with resources, and where equality among participants, both peer-experts and health and social experts by training, are essential. One of the experts of experience describe the essential-ness of equality in the following way:

“Here you are a person with resources. Nothing else.” (Heidi, course trainer)

One participant emphasized that she was met with dignity and equality at the RC, and how being treated that way contributed to her recovery process.

“It was not decisive for me, but it contributed to dignity. That I can walk with my head held high and be me and understand that I have something I can contribute and that I have a value in society.” (Dina, student)

“You are met as an equal person” (Emilie, student)

Underpinning the aspect of equality within the college, one of the course trainers noted that they were all students, regardless of their roles in the RC. They meet as equal fellow people with resources. Citizen meets citizen. In the interviews there are several descriptions of mutual learning processes. One of the course trainers talked about a feeling of neutrality to describe the RC in this regard.

“As a participant and student in college, I’ve never noticed that there are walls, obstacles, it’s just opportunities. There is sort of no restrictions on what we can achieve. There’s nobody coming in and disrupting the processes, so I think that’s important – that it’s a neutral arena.” (Erik, course trainer)

### 3.4. From being alone to being part of a fellowship

The participants talked about a sense of belonging and community at the RC, and that this meant a lot to them. Several of the participants felt that they could not find their place elsewhere, but at the RC, they experienced a sense of belonging and a sense of community they had not before. Among other things, meeting others with the same background in the courses meant that many of the participants felt that they were not so alone in having problems.

“It’s good to hear that other people and…. In a way, yes, not that it’s good to hear that others are struggling… but that I’m not alone in having problems then. It’s been nice or not nice, but it’s been good.” (Grete, student)

They described being in an RC as becoming a group that was mutually engaged in each other’s processes, and that they cheered each other on and gave each other advice. They found that listening to others talk about their own process and getting input from others were very educational and rewarding.

“Yes where… how willing people were to help each other and give each other advice. There was a girl there who was in the orienteering association or something like that, that got us into orienteering courses. And yes… We share ideas and solutions and stuff like that. It became very much that kind of community.” (Bernt, student)

The participants said that the courses were perceived of as safe settings and were set up in a way so that everyone would get to know each other. For several of the participants, social interaction had been something they struggled with a lot previously, and the courses had helped make it easier to get to know new people. In the RC they also got to know people who were very different from themselves and learned to respect other people’s opinions.

“I do not know, you kind of get to know people in such a good way. At least I noticed that the way you talk… It’s no matter what that person is, if it’s a person you would never get along with in the past, you get along now.” (Henrik, student)

One of the participants said that the most important thing that the RC had contributed to in his recovery process was mastering basic social codes, which were tools for mastering meaningful everyday interactions as a normal citizen.

“I honestly did not know how to talk to an A4 person. Eh… It’s something different when you come to a treatment, because they are sort of trained, they have those books and know how to take it. But when you get on a course… Eh… and those in a way… yes, they really taught me the thing about how to talk, how to… active listening. Eh… what is it called… Yes, small talk! Small talk, active listening.” (Henrik, student)

### 3.5. An “inalienable addition”

Most of the participants either attended RC in parallel with or after a period of some other kind of support, either specialist care (outpatient or inpatient) or community services. When they talked about their attendance at the RC, they also talked about timing. Some said that RC participation came at a perfect time, meaning at a time when they actively wanted to do more about their situation.

“It came at a perfect time and gave me the flow and belief I needed to move forward in life and in that recovery process. And yet nothing like that… that I become… that I believe that I can climb the top of the mountain, but that I will have a good life. That’s the whole point. And I’m going to be at peace with myself, and not torment myself with guilt and shame and all that. I will have a dignified and good life. So… I keep going!” (Dina, student)

Another participant said that they realized (retrospectively) that they had gone through a course during a period when it had not been that useful, because it was too early in the process.

“But it wasn’t helpful to me, just that course there. And it was early in the process, early after I was discharged from alcohol and other drug treatment. So I wasn’t sure where I stood in life. So that might matter.” (Dina, student)

One participant described the processes going on at the RC as something happening “afterwards” (after treatment or prison) on the outside. Still, participants emphasized that it was important to communicate to new people that the course demanded that they were motivated and wanted to participate actively. And that some people might join courses too early in their own healing or recovery process.

Most participants described recovery as a process and that joining an RC could be an invaluable part of that recovery process. They also emphasized that traditional both health/social services and other arenas (like RC) are vital. One participant said that being part of an RC was an “inalienable” addition to other kinds of support and help:

“So uh… But it’s going to be two very different things, in a way. All this here [in the RC] is very much like hands on, it’s very accessible and very easy to use. But you can say… um. It complements each other. Because I would have needed the help I’ve gotten in public anyway, so I could not just have this here, but it’s given me something that I’ve never gotten which I may have wished to find… but which I’ve never gotten in psychiatric treatment. But yes, it will be two different things then. I think this is inalienable [*sic*]. Yes, I think it’s good. So yes. Both have theirs, but in completely different ways.” (Emilie, student)

Many of the participants also had experiences of joining various kinds of voluntary organizations and self-help groups that shared elements of the same recovery-oriented thinking and practices that RCs have.

## 4. Discussion

Recovery from substance use and mental health issues is a matter of physical and mental health, but is also dependent on the processes of building a positive social identity through social inclusion and narratives of change (34). Experiences of public stigma, social exclusion, powerlessness, and injustice have for many people been attached to conditions of mental health and substance use difficulties, which in turn might also reinforce those conditions (3).

In this paper we have used the ideas from positioning theory to emphasize the interconnection of societal moral order (storylines), and to demonstrate that while individuals accept certain positions, they also have the capabilities to reposition themselves, introducing alternative storylines in the settings they are involved in (28). In the setting of an RC, within the moral order of this institution, the people attending are actively ascribing to the position of student, in a storyline of learning and equality. For many people, this position contrasts with the position they have been offered in encounters with regular health and social services, where they have been positioned as patient/client/user, in a storyline of being a person in need of care.

The accounts of participants in this study show that they have taken up and appropriated the rights and duties offered in this setting and have thereby entered into positions that contribute to life-changing processes, with enhanced self-worth, less stigma, and more social fellowship. Being part of a RC has enabled new positions in life, as resourceful and valuable citizens, with possibilities for better futures. The degree to which RC contributes varies when it comes to participants’ needs or intentions for repositioning.

### 4.1. Anti-stigma work

Stigma can be defined as an attribute that results in widespread social disapproval, involving both the recognition of differences and devaluation that can occur in social interactions; stigma does not reside in a person, but rather in that person’s social context (2, p. 1). For our purposes, it is useful to distinguish between public stigma (a cognitive representation that people hold regarding those who possess stigmatized conditions) and internalized self-stigma (a reduction of self-worth accompanied by psychological distress experienced by people with a stigmatized condition) (2, p. 3). It has been shown that internalized stigma reduces a person’s hope and self-esteem, leading to negative outcomes and barriers to recovery (35).

In the context of public stigma, our findings indicate that being part of an RC represents an important possibility for repositioning oneself from a stigmatized position (in a stigmatized space, being sick or being “what’s on the paper”) to a non-stigmatized position (that of student or fellow human). In the setting of an RC, a person is more than their addiction issues or psychological difficulties – they are not their diagnosis, they are an entire person. In addition, our findings suggest that RCs can contribute to increased public knowledge of substance use and mental health issues, which presents alternative storylines for the local community. This occurs as local stakeholders, such as voluntary organizations and public services, are engaged with the college, and the college actively extends its outreach by delivering educational sessions to university students and various service training programs. These actions provide meeting points for different kind of actors, promoting and providing information about RC in different channels. Being a community-based RC (13) might amplify the possibility for this kind of anti-stigma work.

Similarly, in the context of self-stigma, our findings show that being part of an RC contributes to an inner (intrapersonal) repositioning, from having a very low sense of self-worth to being a person of value and resources. These processes are embedded in the social interactions that take place at an RC, representing equality, recognition, and dignity. A person’s institutional (re)positioning as a student, and the intrapersonal (re)positioning as a person of value, mutually reinforce each other and contribute to life changes. Other studies have also highlighted the importance of working with internalized stigma (18, 36). Thériault et al. (18), in their systematic literature review, point out that internalized stigma is only assessed with a standardized tool in one study, and that more research could be useful for examining this central aspect of social recovery. The qualitative findings from our study support the conclusion regarding the importance of investigating this perspective further.

Muir-Cochrane et al. (24) described the RC as a transition space from an identity as excluded and a patient to an identity as normal and a student, which aligns very well with the findings of this study. The concept of positioning ads to these findings in theorizing about how societal discourses or storylines interact with personal identity processes.

### 4.2. Being in a responsible position and barriers for attending

One of the findings in this study is that a person shifts from being a passive recipient of care to an agent actively responsible for life changes – from the position (as some of the participants put it) of being a victim to the position of being responsible for one’s own life and making desirable changes. Participants describe this process of repositioning as crucial to their recovery process. This new internalized position also aligns with the institutional positioning of the participants as students.

At the same time, to some degree, this process can also be a barrier for attending the college. The repositioning as active agent, inherent in the social setting of an RC, can also have a disadvantage that it creates new demands on individuals in the form of higher expectations. A storyline of equality and learning involves certain expectations about how persons should act within that context. One must be in a position where one is able to take the responsibility, and some potential participants might not be in a mental condition amenable to these expectations. One can be referred to regular health and social services (which might be exactly what a person need), but one must choose to attend an RC.

Questions about potential barriers for attending RCs have been much discussed (20, 37, 38). As a starting point, RC is open to everybody. Still, some studies propose that attending RC would be most helpful for people at certain stages of recovery and that an RC is less useful for a person experiencing acute illness (37, 38). Another barrier could be connected to low a sense of self-worth and a lack of belief that one has something to contribute (39).

These barriers were touched upon by the participants in this study when they talked about timing: when they were ready for the RC or not; when their experiences were that they started attending courses to early in the process; that attending RC is something that happens “afterwards”; that it is important to communicate that an RC requires active participation. The discussion of timing and barriers are also related to how places like RCs can be seen in relation to other health and social services. It is important to discuss whether existing barriers are the way an RC should be, or if some barriers should be lowered.

### 4.3. An invaluable addition

Although the participants in the study emphasized the vital importance of attending RC as part of their recovery process, with the built-in possibility for repositioning, they still emphasize that regular health and social services play important roles. One of the participants described RC as an “inalienable” addition, which points to how highly she valued RC while at the same time noting that it is an *addition*. That is, there is a time for regular services and a time for places like RC, and the two things complement each other. It is also important to remember that the participants also mentioned other peer-focused services and organization that serve many of the same purposes as an RC does. Thus, when in a recovery process is RC useful? And how can RC and regular services interact in a way that is most useful and sustainable for the citizens they serve?

These kinds of interactions take place in the very co-creation of courses in RC by people working in the services and the taking part in the courses. Both community services and specialist health care services incorporate recovery-oriented perspectives and interventions, and solutions like hiring peer-workers is high on the agenda (40). Still, specialist health care remains a context in which the bio-medical model prevails (41). What can services learn? Our study suggests that it is important to be aware of how traditional health and social services position people in encounters, and that there needs to be a repertoire of ways to meet people in accordance with their current needs and where they are in their process of getting better.

## 5. Conclusion

The study highlights the vital importance of providing alternative arenas like RC for enabling work with life changes, as an invaluable addition to regular services when it comes to the challenges of substance use and mental health issues.

## 6. Limitations

The generalization of the study is limited by its relatively few participants from two RC locations in Norway. Another limitation is that course participants were recruited solely from one of the colleges, due to practical circumstances and resource constraints. It was hard to recruit participants who attended courses, so the sample might be biased regarding their opinions about the RC and its significance. Course trainers with background rooted in personal are recruited from both colleges and are therefore more representative. The study lacks the perspectives of course trainers with professional backgrounds, which would have added to the perspectives on the processes taking place during the study. This perspective will be elaborated on in an upcoming publication.

## Data availability statement

The raw data supporting the conclusions of this article will be made available by the authors, without undue reservation.

## Ethics statement

To protect privacy for the participants, the project is processed and recommended by NSD, Norwegian Centre for Research Data (now SIKT, Norwegian agency for shared services in education and research), in accordance with applicable legislations (nr. 689,039 and nr. 660,505). The Data Protection Officer at the University of Stavanger appraised that the project was properly carried out, and that benefits and risks are assessed against each other in accordance with the Guidelines for Research Ethics in the Social Sciences and the Humanities (27). The participants provided their written informed consent to participate in this study. The project was not considered for ethical approval at the Regional Committees for Medical and Health Research (REKs), as the project falls outside the scope of the Health Research Act from 2009.

## Author contributions

AS, LK, RN, and IR contributed to the conception and design of the work. The first part of the analysis and interpretation of data was conducted by LK and RN, with critical revision by AS, AK, and IR. The second part of the analysis, across groups, was conducted by AS in cooperation with LK, RN, AK, and IR. The article was drafted by AS in cooperation with IR, and critically revised by the LK, RN, and AK. All authors contributed to the article and approved the submitted version.
